# 1217. Game of Trophs: *Stenotrophomonas maltophilia* Management 2.0: Before and After an Antimicrobial Stewardship Intervention

**DOI:** 10.1093/ofid/ofad500.1057

**Published:** 2023-11-27

**Authors:** Andrea H Son, Michelle T Hecker

**Affiliations:** The MetroHealth System, Cleveland, Ohio; The MetroHealth System, Cleveland, Ohio

## Abstract

**Background:**

IDSA guidance on the treatment of *Stenotrophomonas maltophilia* infections version 2.0 recommends caution in the use of levofloxacin, reserving its use as a second line option for mild infections and using it only as a component of combination therapy for moderate to severe infections. The use of ceftazidime is not recommended. In July 2022 we implemented a multifaceted intervention including suppression of levofloxacin and removal of ceftazidime susceptibility reporting in the electronic medical record, education to ID providers, and prospective audit and feedback (PAF). We herein describe *S. maltophilia* treatment and outcomes before and after this intervention.

**Methods:**

This was a retrospective single center cohort study including all patients from whom *S. maltophilia* was isolated in culture between 7/1/2021 – 6/31/2022 (before) and 7/1/2022 – 3/31/2023 (after). The primary objective was to describe the rates of adherence to the recommended use of levofloxacin and ceftazidime for each period. The secondary objective was to describe clinical outcomes.

**Results:**

One hundred eleven patients were included in the study. The respiratory tract was the most common culture source. Levofloxacin susceptibility data were visible to providers in 100% and 2% of cultures in the before and after periods, respectively. Ceftazidime susceptibility data were visible to providers in 20% and 7% in the before and after periods, respectively (table 1). ID was consulted more often in the after period (30% before vs 47% after). Adherence to recommended treatment overall was greater in the after period. Fewer patients received levofloxacin monotherapy and no patients received IV ceftazidime in the after period (table 2). *S. maltophilia* was isolated in subsequent cultures in 12% and 13% of patients and levofloxacin resistance developed in 3% and 2% of isolates during the before and after periods respectively (table 3).

**Figure:**
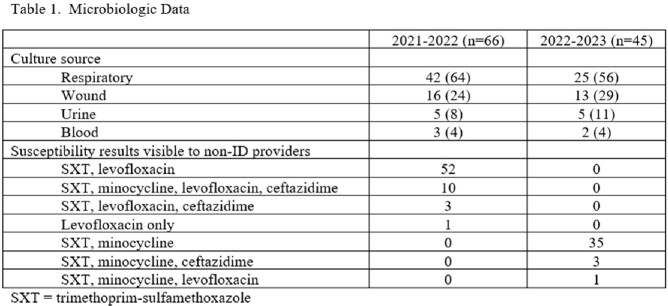

**Figure:**
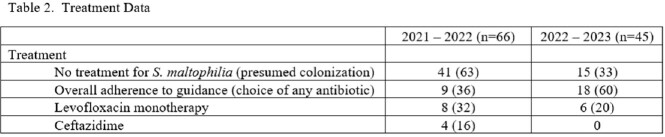

**Figure:**
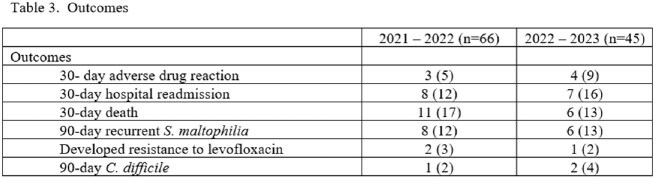

**Conclusion:**

A stewardship intervention including changes in susceptibility reporting, education, and PAF was associated with an increase in the percentage of patients receiving appropriate therapy. Clinical outcomes did not change.

**Disclosures:**

**All Authors**: No reported disclosures

